# Digital capture of fingerprints in a disaster victim identification setting: a review and case study

**DOI:** 10.1080/20961790.2018.1521327

**Published:** 2018-11-13

**Authors:** Bryan T. Johnson, John A. J. M. Riemen

**Affiliations:** a Federal Bureau of Investigation, Quantico, VA, USA;; b Dutch National Police, Zoetermeer, The Netherlands

**Keywords:** Forensic sciences, disaster victim identification, postmortem fingerprints, digital fingerprint capture, INTERPOL DVI, ridgeology

## Abstract

Identification of victims following a mass fatality is conducted by collecting and analysing a series of scientific identifiers and contextual information of each decedent. Recently, there has been a paradigm shift demanding that this complex identification process be accelerated to meet the needs of the surviving families, politicians and even the media. Postmortem fingerprint identification is a fast and efficient means of victim identification, and through the use of new advances in technology, the digital capture of fingerprints in a disaster victim identification (DVI) setting will play a strong role. This paper provides an overview of current technology and explains how this technology can adapt to current DVI procedures. The Malaysian Airlines Flight 17 (MH17) incident is a recent example of a DVI event that utilized new digital fingerprint capture technology and further demonstrates why such technology is warranted in future mass fatality operations.

## Introduction

### Background on fingerprints in disaster victim identification

Disaster victim identification (DVI) is a term that is used to collectively describe the process in which victims of a mass fatality are identified postmortem (PM). Depending on the locale of the DVI event and the countries who are working on the incident, the number of victims involved could vary from one to many. The term “disaster” does not imply a specific number of victims but rather implores the use of scientific and contextual clues to effect victim identifications. What is consistent between entities performing DVI work is the desire to properly identify victims to the greatest extent possible, using any viable means. While most countries equipped to handle a mass fatality have their own standards and protocols for performing DVI work, in 1984 International Criminal Police Organization (INTERPOL) published a DVI guide to ensure that proper identification techniques are used and to promote universal practices [1]. The current guide was last revised in 2014 and continues to outline the currently suggested practices in victim identification.

Victim identification is also a legal matter, and the final issuance of verified identification can only be completed by the presiding legal authority [2]. When attempting to identify a singular decedent who was found in a controlled environment such as their own home or workplace, it is not uncommon to assume identity using only contextual information. This could be a description of clothing the person was last seen in, or an identification card found in a purse near the body that looks similar to the victim. While this may provide a tentative identification, it is easily subject to error and the forensic community has moved away from relying on such contextual clues [3]. In the case of a disaster event or when the decedents are completely unknown, it is imperative that primary identifiers be used for identification. Primary identifiers for DVI work include fingerprints, dental and DNA. These primary identifiers can be supported by secondary identifiers such as tattoos, scars, medical devices or other physical distinguishing features [1]. Either in an open-population event such as a bombing or hurricane, or in a closed-population event such as a plane crash with fragmentation, the singular use of contextual information will often lead to false associations of identity. There have been many mass fatality cases all over the world in which one decedent was carrying the identification of another individual amongst their belongings, or two victims looked visually similar, and the wrong family was initially told their loved one was deceased [4]. Wounded casualties that end up in the hospital with head trauma also typically cannot identify themselves and their identity must not be assumed.

The use of PM friction ridge prints (fingerprints, palm prints or footprints) for victim identification is often the fastest primary identification method in DVI work. Their use in the identification of a decedent is no different than identifying a live person. Additionally, fingerprints are the most commonly used biometric identifier and have been seamlessly integrated into daily life. They are relied upon at borders, police stations, amusement parks, by employers wanting to verify the identity and the background of applicants, to unlock smart phones, and allow access to controlled facilities [5]. Collection of antemortem (AM) fingerprints for visa and passport databases is also growing rapidly worldwide. These, and other fingerprint databases, can also be searched against when the DVI victim is unknown. As the use of biometrics expands worldwide, obtaining AM fingerprint records for an individual is becoming easier and faster than ever before.

Identifications via other primary identifiers bear different challenges. In order to use dental records to identify a victim, you must have a presumed identity of the decedent so that AM records can be located. Obtaining dental records and knowing who to contact for medical information can be challenging especially in cases where the entire family may be victims in the disaster. DNA samples obtained from a decedent must be further developed into a profile, which then requires a known or familial AM comparison sample for identification. The difficulty of familial DNA testing is that two children of the same sex and from the same parents cannot be distinguished. Two decedents in one DVI event could be associated as being related, which may offer investigative value, but it would not allow for individualization of the remains. In any case, identifying a victim quickly so a family is not left waiting can be done even with fragmented remains. It requires the priority be to first establish identity and then to later fully examine and re-associate all available remains. This is an alternate approach to standard mortuary procedures and can offer the chance to triage and assess which (mostly intact) bodies can be easily and quickly identified by ridgeology and which bodies need further in-depth investigation to determine the identity using slower modalities such as DNA or dental examination.

The collection of PM friction ridge prints may have their own complications. In cases where victims were exposed to extreme heat or result in high fragmentation where no limited usable friction ridge skin is present, there is little or nothing that the ridgeologist can provide. Assuming that a victim has enough viable friction ridge skin remaining, PM fingerprints can be recorded in a variety of ways. Analogue methods such as ink and paper or fingerprint powder and adhesive labels are often the standard techniques for recording friction ridge prints in most countries. Digital capture methods such as the use of a fingerprint scanner are being used more frequently and instantly create a record that can be searched and/or compared against AM databases. Many of these digital capture devices are on mobile platforms making them ideal for DVI work. Digital photography can also be used to capture the friction ridge skin when it cannot be recorded/captured physically. The condition of the decedent will generally dictate the method of capture, but the end result is the same, a record that can easily be used to quickly identify mass casualties.

### Role of the ridgeologist in DVI

In an organized DVI process, a mortuary will often be converted or re-constructed for a more efficient processing sequence. In this assembly line style processing, the decedents move through a series of stations, each dedicated to a specific component of PM examination (i.e. photography, radiology and odontology). There will typically be a fingerprint station early on in the process in which PM prints are collected to be compared at a later time. Simultaneously, investigators outside of the mortuary operation are attempting to locate AM biometric records and contextual clues that can be used for comparison by interviewing victims’ families and populating missing persons’ reports. The study and use of the uniqueness and persistence of friction ridge skin (fingers, palms and feet) for identification purposes are commonly referred to as Ridgeology [6]. A ridgeologist is the practitioner that collects and compares known and questioned friction ridge prints to effect an identification (or individualization). Depending on the work being performed, this role may be divided into two or more parts, especially in a DVI setting. Experienced technicians or other trained individuals may be employed to capture and record the prints, leaving the qualified ridgeologists to conduct the comparison of the records.

The level of work and type of processing required by a ridgeologist in a DVI response is often directly related to the type of mass fatality incident. A mass shooting incident typically allows for straightforward and standard PM fingerprint collections as bodies are largely intact and will likely have minimal damage to both hands. A bombing or a plane crash however, creates a multitude of issues where hands or feet may not be present to print, or multiple advanced processing techniques may be required to rehydrate the skin so that a recording can be taken. Some PM processing can be very time consuming and requires a different level of expertise and equipment. There are techniques such as osmotic rehydration for macerated remains or sodium hydroxide rehydration for mummified remains that can assist in making damaged friction ridge skin recordable [7,8]. A ridgeologist may be needed for these difficult cases to not only know how to best recondition the remains but also to capture high quality PM exemplars. Once the friction ridge skin has been reconditioned, a variety of techniques may be used to record the prints.

The final and most important role of the ridgeologist within DVI is the comparison of AM and PM data. A qualified ridgeologist examines all of the information present in a PM record and determines if it is of suitable quality for comparison. If deemed usable, the record may be compared to known AM records obtained by the AM investigators or they may be submitted to fingerprint databases for searching. The results of the comparison or of the automated search are documented, and the results are presented to the reconciliation board. A reconciliation board typically consists of representative experts that handle the final identification process, wherein all of the contributing information identifying a person (i.e. ridgeology, odontology, DNA, secondary identifiers, contextual property) is presented, and a decision is rendered by the presiding official.

### Types of digital fingerprint capture technology in DVI

There are many different types of digital capture fingerprint devices and platforms on the open market. They are available in most languages and operating systems, typically customized to the end user’s needs. Some of the devices are meant for authentication, such as a smartphone unlocking sensor, while others are designed for capturing a full fingerprint record of an individual, often referred to as a “livescan” system. In addition to different styles of readers based on intended use, there are also a variety of fingerprint scanner technologies [9]. These include optical, capacitive, thermal, ultrasound, radio frequency (RF) and pressure-based scanners [10]. Some newer scanners employ multiple types of technology all within one scanner, such as capacitive and optical, in order to obtain better results or to combat spoofing. There are lower-tech options, such as smart phone cameras, that may still expedite the process of transmitting photographs taken of analogue fingerprints for identification [11]. New innovations are often seen in emerging technology, such as ultrasonic scanners, which were developed to combat spoofing of sensors and issues with contaminants on the surface of the skin. Most (if not all) of the scanners were designed to work with the living, and this may cause problems when choosing a scanner technology for PM prints.

Optical scanners are the most commonly used law enforcement capture platforms. They have the most robust development since they have been used the longest and are the basis behind most traditional “livescan” systems. Optical scanners work by reflecting light off of the finger that has been placed on an optical plate and use a computer sensor to read the differences in contrast among the ridges and furrows to create a two-dimensional representation of the fingerprint [12]. Depending on the model, some are easily affected by liquid and/or debris on the finger, which is commonly found with the deceased. Another issue with optical scanners is that they are sensitive to residue left behind from previous scans, which may appear in each successive image [10]. When working with dirty or wet skin, as is commonly found when working with the deceased, by the time the whole hand is printed the final recordings may be obscured unless the scanner is consistently and thoroughly cleaned in between recordings.

Capacitive scanners rely on electricity and the skin’s ability to allow electric current to pass through the cells. The difference in electrical current from where the ridges touch the surface of the scanner versus where the furrows do not, allows the scanner to detect where the ridges are [12]. These scanners are becoming more common because they are not as easily spoofed as some optical scanners, which can be fooled using an image of the fingerprint on the scanner. For PM printing, the specific capacitive technology design is important. Many of the capacitive scanners require that the person is alive and passing electricity through their body. The body naturally is grounded when living, permitting the required flow of electricity necessary for a capacitive scanner to function, however this electrical connection is not sustained with a deceased individual. In order to make the scanner function, a live person could concurrently touch a part of the scanner without gloves, but this is not good practice in a biohazard environment. Some scanner types also feature “live detection” technology to keep the scanner from being spoofed [13]. Unfortunately, these scanners then cannot be used with the deceased unless that technology can be disabled.

Another type of scanner that is not conducive for PM fingerprinting relies on skin temperature. Thermal scanners use the minute differences in heat produced between the ridges and furrows of friction ridge skin coming in contact with the scanner [12]. There are typically no thermal differences when working with the deceased, so these scanners cannot be used for PM printing or DVI work.

One of the newer technologies to the field, RF scanners, is promising for PM printing because it isn’t as affected by contaminants, damage to the outer layer of skin, electrical conductivity or body temperature. RF scanners, while also a type of capacitive scanner, relies on RF signals which are injected into the skin and then read by a detector array. These signals penetrate the outer layer of skin and bounce off of the lower dermal layer of skin before being read by the array [10].

Ultrasonic scanners use the same principal as RF scanners in that ultrasound waves are bounced against the dermal layer of the skin and are read by a chip [12]. These scanners are designed to create a three-dimensional image of the fingerprint, which then can be converted into a two-dimensional image for comparison [14]. These are one of the newest styles of scanners to be developed and have great potential for PM printing since they also can read below the surface of the skin, eliminating contamination interference. Companies such as Qualcomm^®^ are using these scanners in mobile devices for user authentication because they are very difficult to spoof.

The leading technologies often employ more than one type of scanning modality, either to improve accuracy or security. The application usually revolves around security or personal authentication, not around printing the deceased. The three main factors affecting DVI work and PM scanning are size, manoeuvrability, and the ability to print a non-living person. Most of the current systems are designed such that a live person is manipulated against a fingerprint scanner, like with a criminal booking station. The size and shape of the scanner platform is ergonomically designed to print a live, standing person. They are usually large and sturdy, meant for high throughput and are tethered to a computer system. Recent mobile platforms have partially removed this limitation, but the digital scanners most often used for recording a full fingerprint card are still bulky and difficult to move around. Additionally, when printing the deceased the scanner must be manipulated against the decedent, rendering many stationary systems unusable [3]. A large and heavy scanner cannot be easily manipulated against a gurney while still tethered to a computer. As previously stated, decedents are often in rigour mortis at the time of printing, which causes all of the limbs and digits to stiffen. This can add to the difficulty of PM printing, without the addition of a large scanner.

The capture technology selected is just as important as the transmission capability. The added value of capturing PM prints digitally is to be able to quickly transmit them to a regional fingerprint database that can search and match them to AM records. Some of the commercially available systems will even transmit the collected records to the proper authorities and facilitate a search response through designated secure portals. This can result in real-time identifications; helpful for expedited victim or criminal identification. If multiple countries are involved in the incident, digital records also allow for quick transmission abroad. While analogue cards can be scanned on a flatbed scanner and sent digitally, using a high-quality digital capture device will save the time required to do that. It is important to know the file format each scanner outputs, so that the end user will be able to receive and view it. If there is a proprietary file format that requires special software and decoding, it will negate the benefits of using the scanner.

### Role of digital fingerprint capture in DVI

There is a more recent paradigm shift in the DVI process that is being driven by the current social climate that expects immediate results. Years ago, processing a fatality incident could take months and the only media coverage was via television and possibly a weekly newspaper. With more recent events, and the multiple social media platforms accessible on most mobile devices, details of an incident can be found on the internet within minutes, or even live streamed. Within hours, the names and faces of victims assumed to have been killed in the event are being run on international media outlets and the criticism of the length of time the DVI process is taking begins. Families are notified by the media long before officials have had a chance to conduct any scientific examinations. There also is an evolution of the victim’s family members becoming more outspoken. In the Malaysia Airlines Flight 17 (MH17) incident, for example, a foundation was established by the “surviving relatives of the MH17 victims”. The foundation is intended to support all surviving family members inside and outside the Netherlands and aims to help with grief coping, commemorating of the victims and ensuring the best interests of the surviving relatives [15].

In both criminal events, such as the mass shooting in Las Vegas, NV [16], or accidental incidents, such as the Grenfell Tower fire in the United Kingdom [17], media outlets are quick to start questioning the speed of the DVI process. There is also what is known as the “CSI effect”. In 2006 according to the Nielsen ratings, almost 100 million people viewed television programmes that centred on scientific evidence in criminal cases. “CSI” was one of the most watched television programmes. This has resulted in lay persons having unrealistic expectations for the use of scientific evidence [18]. The biggest challenge facing the forensic science community is explaining that the speed of forensic investigations in “CSI” is simply not realistic. Any DVI operation is a sensitive one, especially when communicating with the surviving family members about how long it may take to find out the status of their missing loved one.

The necessity of the immediate capture of biometrics is often debated. It is simple to suggest that all victims should be fingerprinted as they are found and recovered, and then the identification process is jump-started. Unfortunately, especially in the case of criminal mass fatalities, identification of the deceased is only one small part in a much larger investigative process. Likewise, not all disaster victims can be identified with fingerprints immediately. Before any of the bodies are moved or handled, other evidence must be preserved and collected. The scene must be documented thoroughly and the positioning of the bodies marked. Even if fingerprints were collected on scene, the tracking process of each body is very important so that a victim is not identified on scene and then re-processed back at the mortuary from the beginning. Scene investigators use a different numbering or tracking system than coroners. Different countries may present jurisdictional issues, whereas one entity may control the custody of the bodies and another may control the scene and surrounding evidence. The ridgeologist may not be directly employed by the entity who has custody of the bodies and therefore cannot simply just start printing the deceased. There is not necessarily a right answer or system, each DVI event is incident-dependent, and requires a discussion amongst the DVI team prior to an incident to develop protocols. It may only be necessary to try and identify any possible criminal subjects on scene for intelligence needs and then to process the rest of the victims later. There is often the mentality by the medical examiner or coroner that the victims deserve to all be processed and identified prior to any work being done on the possible criminal subjects. Ethically, the argument makes sense, but this may hinder further investigations into finding other criminal conspirators or even preventing a secondary attack. In a natural disaster incident there is no criminal component so on-scene prioritization may not be relevant. In these cases, victims are processed as they are recovered and identified over time using available resources with prioritization focusing on preservation of the bodies to slow the decomposition process.

All accredited and regulated government mortuaries have standard operating procedures. Most of these facilities will also have a plan for large volume processing. A “large volume” event to one facility may be drastically different than the next depending on the average daily case load. This upscaling effort may simply require calling in additional staff, or it may be more drastic and alter the entire sequence that the remains are processed. It is important to have this distinction, because executing a daily protocol to triage a mass fatality will usually result in chaos and the facility becoming quickly overwhelmed.

The goal of any PM examination is to identify the decedent and to determine the cause and manner of death [19]. In a typical pathological examination, the decedent is processed from beginning to end, to include identification and autopsy, with elaborate notes and documentation to be able to properly fulfil a death certificate. This examination process works for standard day-to-day cases, but will not satisfy the victims’ families waiting in the lobby or at the family assistance centre, nor the politicians and the media covering a disaster event. The main focus of the mass casualty recovery and processing has to be identification. Primary identifiers, whether they be fingerprints, dental, or DNA may be utilized and prioritized prior to any full autopsies, unless circumstances warrant the need. This will likely be a departure from the daily standard operating procedure, but it will be the fastest and most efficient way to identify victims and notify their families. Naturally this is more challenging when there are fragmented remains versus intact bodies, but the end goal must remain – to efficiently identify and notify. The role of digital capture of fingerprints, digital CT scans, as well as the development and validation of rapid DNA [20] is not just to make the identification process easier, they all help to quickly provide closure and to expedite any potential investigation that may ensue following the event.

How fast is fast enough when reporting identifications? The answer may differ among DVI team members, management, the media and families of the decedents. The quicker the process, the greater the risk there is for errors to occur. When less information is gathered prior to vetting an identification, the greater the chance that something may not add up at the end, or a quality measure is missed. The continued emergence of these new technologies should be seen as tools to attempt to keep up with the expectations of those not involved in the process. Victims’ families, politicians, and the media should not dictate the work, nor the rate at which it is performed, but there is rarely a mass fatality recovery effort that doesn’t start with a high-level person on the news explaining how the process will progress. It is the reality of these types of events coupled with the expectation of instant gratification that makes processing a disaster ethically challenging. No one wants to appear in front of a distraught and anxious crowd simply to inform them that there is no definite timeline for completion.

Mass fatality events are not confined to one geographic, socioeconomic or politically charged region. Likewise, jurisdictions that preside over a DVI operation will often have different requirements for rendering an identification based on the city, state or country’s laws. It is important on the first day to understand and establish the requirements for identification based on the locale, especially when multiple nationalities are involved in the incident. One country may require a point standard for fingerprint identification whereas another may employ holistic approach. Other countries may require two primary identification methods before effecting an identification, whereas others may allow for visual and contextual identifications to be officially reported. Furthermore, the type of incident may impact what level of information is needed to effect a conclusive identification. A small, closed-population incident of a few deceased with tentative contextual identifications may not need as rigorous of an approach as an open-population incident with no known victim list. Or, the other option is the same standard of identification may always be required to ensure there are as many quality measures in place to combat the potential for error. This decision may be dictated by legal requirements in the country processing the incident, which may or may not allow for different adaptations based on the given population of deceased. Regardless, the DVI work will be bound to local legislations during the processing of a mass fatality event, and should not be modified to make the DVI process is easier.

Ridgeology comparison requirements and standards differ around the world. International standards exist for the capture and collection of biometric records but these do not dictate how those records are utilized [21]. Once the records have been collected, the ridgeologist conducting the comparisons is restricted by their own country’s laws and standard operating procedures. There are different approaches to print comparison, whether it be a numerical point standard, a holistic approach, or even may include additional quality assurance measures such as verification or blind verification. There is no international standard for establishing sufficiency to declare a match [22]. This is why it is so imperative that the ridgeologist knows the policies for the country performing the work, so that they can relay that to the chief investigator. When comparing a full fingerprint record, there are two routes of identification. In many countries, a full fingerprint record will be searched against a database, and if that computer system assigns a high enough quality threshold, it can effect a “lights-out” identification. If the record is of low quality or only a few fingers are present, there is not enough information for the computer to make a match and a qualified ridgeologist will have to conduct a manual search within the system or do a manual comparison. The “lights-out” method is typically used when submitting prints through a digital capture device to a government database and therefore explains how identifications can occur so quickly. The manual process, regardless of the country and comparison standard, will never have instantaneous results. Having a qualified ridgeologist available is necessary, especially with low-quality records.

All applicable laws, policies and standards must be met to establish a match using the prints, but the final decision lies with what can then be done with that information. Many countries rely on a single fingerprint to verify identity, such as those on passport documents or driver’s licenses. It must be decided that if these single fingers are sufficient legal documents for border entry, they may also be sufficient for reporting an identity to a family. There are many reasons why having multiple levels of identifiers is better than using a singular one, but if that match comes from a primary identifier (especially if all 10 fingers are present), making the decision to release the identity to the families may make the difference in being “timely” versus being accused of unnecessarily delaying the process. Even after the identity is reported, the full gamut of examinations and pathology can and likely should be conducted. The case and the families deserve that. If a digital capture device is used and an identity is produced through procedural processing, it is negligent of the investigator not to expedite reporting that information.

## Case study – MH17

On July 17 2014, MH17 was intentionally shot down over the East Ukraine. The flight was on its way from Amsterdam (the Netherlands) to Kuala Lumpur (Malaysia). On board were 283 passengers and 15 crew members [23]. As soon as the media began reporting that flight MH17 was missing, there was immense pressure to release information about the possible victims from the flight manifest. The immediate demand for information regarding the identities of the victims from the next of kin, politicians, worldwide media and others was conflicting with the traditional DVI principals to provide accurate information only once properly vetted.

### Victim identification – PM

The passengers aboard MH17 included citizens from the countries of Malaysia, Indonesia, the Philippines, the UK, Australia, New Zealand, Germany, Belgium and the Netherlands. Because the majority (196 passengers) were of Dutch origin, a DVI team from the Dutch Police organized the victim identification process in cooperation with DVI teams from all of the countries that had victims on board. In the city of Hilversum, the Netherlands, a morgue was created on a military base and five identification lines were readied to process the bodies for the PM investigation. At that time, the Dutch Police had already developed a “dead-scan” solution for recording digital PM friction ridge impressions. This was originally introduced to support or replace the traditional recording of friction ridge impressions from bodies via ink and paper. The dead-scan was still considered a proof of concept device at the time MH17 occurred but was already in beta-use within the forensic investigation teams of the Dutch police. The dead-scan system utilized specialized software the Dutch designed to support the recording of PM fingerprints. It provides practitioners with several options of scanner drivers and devices for various digital capture requirements. The most compatible devices vary in recording image quality from 500 pixels per inch (PPI) single finger scanners to 1 000 PPI full finger and palm scanning capabilities. The main benefit of the Dutch dead-scan system is that the practitioner has the ability to record multiple flat or rolled images per finger without applying any chemicals or powders to the friction ridge skin. With traditional collection methods the chance of damaging the skin is higher, since you have to apply ink or powder to the skin and clean it off again in order to record each new impression. Likewise, with the traditional methods, it is difficult to manipulate stationary bodies, often in rigour mortis, against cardstock to record quality impressions. A significant advantage to the Dutch dead-scan system is how the small, single finger scanner can be rotated around the finger, similar to how a practitioner manipulates the finger when printing a living person.

Currently, only flat finger scanning devices are being used. The software developed to capture friction ridge impressions allows the operator to record multiple (up to hundreds) flat images of the same finger in various positions by repeatedly rolling the finger around on the plate. A ridgeologist may then decide which image(s) are best for comparison to the available AM records, or choose the most suitable print(s) for searching through an automated fingerprint identification system (AFIS). The ability to record multiple impressions is particularly valuable since most AM fingerprints are not typically recorded by ridgeologists, but rather by minimally trained civil servants. Unfortunately, many AM records may be of poor quality, or are not fully rolled recordings for each finger. For example, an individual who routinely collects prints for visa applicants is often times just recording one plain impression per applicant. This individual may not possess the experience or training to compare prints, and therefore is not concerned with obtaining the most complete recording possible. This is why it is so imperative that technology like the Dutch dead-scan is able to record and retain hundreds of images in a short amount of time. It provides the practitioner with a variety of recordings, further increasing the chance in finding a match to AM recordings.

Following the MH17 crash, the decision to use of the dead-scan system was thoroughly discussed by the PM Ridgeology collection team. Although it had not been tested at this scale before, it had promising potential in adding efficiency to the DVI process. Dead-scan systems were designated for each of the identification lines, and one specialized station was converted into a mobile platform so that it could move about as needed. Three ridgeology experts familiar with the system were deployed within the mortuary to oversee the recording of the PM prints. They floated between the different collection lines to assist with the more challenging cases and assess the quality of the prints that were being collected. This provided instant feedback to the DVI team members and acted as a real-time quality assurance measure. Because the identification lines were staffed by DVI members from different international DVI teams, the decision to employ traditional collection methods or the dead-scan system was left to each of the teams. It was crucial that the DVI teams could work with a procedure they were already comfortable with, rather than add undue stress and increase the potential for errors.

The processing lines were organized so that all bodies and dissociated remains were first photographed. All clothing and jewellery was then removed and separately photographed. Following this documentation process, the PM examination began with the recording of the fingerprints (or other relevant friction ridge skin), DNA sampling, PM dental X-ray, and a detailed full body description. The process ended with a final quality check.

All of the digitally collected PM prints were stored within the dead-scan software using the unique PM number assigned to each set of remains. One significant advantage to this software was that the images were already formatted in the National Institute of Standards and Technology (NIST) file format and readily transmittable to outside agencies. The NIST (ebts) file format is readable by most fingerprint software systems, regardless of language. Any friction ridge recordings that were manually collected with the analogue or traditional methods then had to be digitized and converted before they could be processed or shared, which was a much more time intensive process.

AM investigators were able to use the flight manifest from MH17 to gather information from the airline in order to track down the next of kin. The passengers and crew were from many different countries, so this was a slow and tedious process. The MH17 incident did however, have some unique investigative benefits. The advent of social media platforms provided investigators with a new avenue for AM data. There were many instances where “selfie” pictures were taken by the passengers while still at the airport before or even after boarding that were posted to social media sites. Many of these pictures were publicly available or made available by the victims’ families and provided important contextual information regarding clothing and jewellery that the victims were wearing leading up to the incident. This information was helpful in narrowing down the candidates for comparison to the AM primary identifiers, in order to make conclusive identifications.

AM fingerprints were obtained from new non-public sources for this incident. Dutch passports for example, contain two fingerprints of the owner embedded digitally in an encrypted chip within the booklet. The Dutch Police had only started working with these fingerprints less than a year before the crash. They had previously been used in a few cases for the identification of an unidentified body, but proved extremely helpful in the AM collection process for MH17. One challenge with the passport prints was that the physical passport was needed in order to extract the fingerprint record, and the chip had to remain intact. Because of the high heat and fragmentation that results from such a crash, some passports were not located and others were badly damaged and unable to be read. Other non-public sources of AM data were obtained from the Indonesian and Malaysian authorities. They provided the DVI team with excellent AM facial photos, fingerprints and even iris-scans for all of their victims pulled from their national database. As of 2012, Indonesia proved to be the most advanced user of electronic identification (eID) credentials with biometric-enabled identification, having collected this information from all of its citizens [24].

The US Department of Homeland Security (DHS) biometric database was another important AM fingerprint source. It maintains biometric information for over 200 million travellers from all over the world [25]. While only one of the passengers on board possessed dual American–Dutch citizenship, many of the passengers had visited the US at one point or another for business or vacation. By creating a secure connection with the US Federal Bureau of Investigation (FBI), assistance from DHS and the FBI allowed records to be extracted and transmitted to the AM investigators. This was especially important for the Dutch victims where no passport was found or the chip was severely damaged, and no other biometrics were readily available.

The last source of AM fingerprints for the victims of the MH17 incident were their individual residences. When no AM data can be obtained from logical government sources, it is possible to go to the home of the missing person and collect items that they may have touched. These items can then be forensically processed to develop latent prints that may be used for comparison to the recorded PM prints. Unfortunately, locating personal effects that have been touched can prove to be a difficult task, since people have the tendency to clean their house before they leave for a trip. AM investigators will typically try to locate items like dishes and drinking glasses that were used a short time before the victim left for the airport. Crime scene investigators are traditionally responsible for the search and recovery of items that may contain latent fingerprints for criminal cases. Searching for items that may possess the fingerprints of a missing person in their own residence is different however, than looking for evidence in a burglary. The MH17 AM investigators had to be briefed and instructed on the best types of surfaces for latent fingerprint evidence, such as personal items like school (paper) notebooks and personal diaries.

### The role of AFIS in *MH17*

Since the early 1980s, AFIS systems have been considered one of the most valuable tools for ridgeologists in searching friction ridge prints through large databases. In the Netherlands, the MH17 victims’ prints were searched against both the criminal and immigration AFIS. A secure transmission connection to the FBI made it possible to utilize the US Government AFIS, by having the records transmitted and searched through the millions of fingerprints they maintain. The Dutch Police also used their AFIS for searching through a small database setup using the collected AM records and for conducting one-to-one manual comparisons on-screen. The Dutch system has a double-blind ACE-V (Analysis, Comparison, Evaluation and Verification) protocol included within the workflow, which provides transparency and quality assurance within the fingerprint comparison process. The decision to create a special AFIS database for the victims was due to the high number of victims and disassociated fragmented remains. There were over two hundred victims on the plane, in addition to the partial remains with significant pressure to complete the identifications in a timely manner.

It was considered unlawful and unethical to use and search the standard Dutch criminal AFIS for this task, so the manufacturer of the Dutch AFIS was asked to clear and then prepare the Dutch Acceptance system for this task. The Acceptance system is one part of the overall national Police AFIS that consists of separate Test, Acceptance and Production systems. With the full AFIS capabilities available, the searching of fingers, palms and even latent prints became available for the ridgeologists. Latent to latent print matches could also be made to tie together AM data that had been collected from the residences. This proved to greatly speed up the comparison process and make it more reliable. With the large amount of data that had to be processed in a very short expectation of time, a human error such as a false positive or false negative conclusion, is more likely to be made. The use of AFIS technology in this setting can help reduce the number of erroneous conclusions [26].

### Results from the MH17 victim identification process

In total, 243 friction ridge print identifications were made from the decedents and dissociated remains. These identifications supported the identification of 151 victims [27]. The process was efficient and streamlined in part due to the use of digital capture on the remains and real-time sharing of digital recordings. The success of the dead-scan system has enabled further development of the Dutch Police’s fingerprint capture capabilities.

## Conclusion

DVI is an intricate and arduous process that involves cooperation through many levels of government, science and politics. The DVI process must continue to evolve to meet the demands of the public, and the growing paradigm shift expecting more expedient victim identification following a mass casualty event. Leveraging available technology across forensic disciplines for DVI use, adapting tools to enhance mobility and embracing alternate capture technology ideal for compromised remains, can improve and expedite current DVI response without impacting quality. Full evaluation of the factors involved with the incident, to include local, and federal regulations, considerations of population, and forensic limitations can further improve DVI response. These factors came together successfully, across international lines, in the utilization of electronic capture and transmissions at the MH17 crash, and are a great example of embracing current technology and future tools for a successful DVI deployment. The ability to share biometrics in real time may prevent a secondary attack. It may prevent co-conspirators from fleeing the area, saving on costly man-hunt searches. The prevalence of biometrics in today’s world, even for the simplest of tasks like unlocking a smart phone or tablet, is an obvious indication that this technology is here to stay and should be utilized to the fullest extent.
